# On Modern Progress in Ophthalmic Medicine and Surgery

**Published:** 1894-10-20

**Authors:** Robert Brudenell Carter

**Affiliations:** Consulting Ophthalmic Surgeon to St. George's Hospital


### On Modern Progress in Ophthalmic Medicine and Surgery.

By Robert Brudenell Carter, F.R.C.S., Consulting Ophthalmic Surgeon to St. George's Hospital.

### INFLAMMATION OF THE IRIS.

It is scarcely possible for anyone, whose medical knowledge is practically limited to the teaching and practice of the last quarter of a century, to form an adequate conception of the sway which was exercised by the word "inflammation" over the minds of many of our forefathers. "Inflammation," with its four attributes of pain, heat, redness, and swelling, was regarded less as a morbid condition to be alleviated than as an enemy to be subjugated, and to be subjugated by measures which, in the language of that day, were sometimes described as "heroic." I lately had occasion to turn to Dr. Bright's original papers on the kidney disease which bears his name, and which were contributed to Guy's Hospital reports about sixty years ago. Guy's Hospital stands alone, I believe, in having from a remote period made provision for the treatment of diseases of the eye; not with any perception, in the first instance, that they should be looked upon as falling within the ordinary province of medical science, and should therefore be dealt with as part of the daily work of the institution, but as the material of a specialty almost distinct from either medicine or surgery, to be practised in the "Eye Infirmary attached to Guy's Hospital." The attachment was, nevertheless, sufficiently close for some records of the work of the Eye Infirmary to find their way into the general volume of reports; and these records are eminently worthy of perusal. It is difficult to say whether the want of precision in the descriptions, or the unnecessary severity of the treatment, may be taken to afford the greatest contrast to the methods of the present day. It is exceedingly difficult to conjecture, from some of the descriptions given, what the conditions would now be called, whether they were acute glaucoma or iritis aggravated by supposed remedies; and it is still more difficult to arrive at any conclusion as to whether the partial recoveries related were in any degree to be ascribed to blood-letting and to profuse mercurialisation, or were merely the triumphs of nature over these depressing agencies. The sole idea seems to have been that the eyes in question were "inflamed," and that the "inflammation," without much regard either to the tissues affected or to the general health of the patient, was to be knocked down by the measures which were then commonly directed against it. Of the results little more is set forth than that some of the patients became quite blind while others did not, and there is nothing to show what amount of vision the latter retained. The precise determination of this important question was not even thought of for many years after the time now under consideration; and one of the leading ophthalmic surgeons of London, in a book published so recently as in 1858, described the ultimate condition of one of his hospital patients by saying that "the man recovered sufficient sight to be able to earn his living by driving a cart over four miles of bad road to fetch coal."

I am disposed to think that the first step towards a proper discrimination between the various kinds of inflammation of the eye, and of the tissues respectively affected by them, was taken by the late Sir William Bowman, and forms not the smallest part of the debt which we owe to his memory. His lectures on the "Anatomy of the Parts Concerned in Operations upon the Eye" had the effect of keeping the several tissues apart in the minds of those who studied them; and of substituting, for the single idea of "the eye," that of the "cornea," the "iris," or the "conjunctiva." The work thus commenced was powerfully seconded by Sir William Bowman's illustrious colleague, Sir Thomas Watson, who dwelt much upon the diseases of the eye in his classical lectures on the "Principles and Practice of Physic," and who used the visible differences between conjunctivitis and iritis to illustrate the invisible but similar differences between bronchitis and pleurisy. We have since come to recognise that the morbid processes which, for want of a better name, are still included within the word inflammation, may be, and often are, limited to particular tissues, and that, when so limited, they are totally different in course, character, and results. The most important of the consequent peculiarities are those which distinguish inflammation of the iris from that of other ocular structures, and which confer upon it, by reason of its possible consequences, a very prominent place in modern ophthalmology.

Of the actual causes of inflammation of the iris, or, to use the shorter and more convenient term of "iritis," we know nothing. The disease frequently occurs as one link in the chain of morbid action which follows syphilitic infection, and it is frequently seen in connection with the rheumatic diathesis. But it also occurs independently of these constitutional conditions, and we are absolutely in the dark as to the manner in which either rheumatism or syphilis is concerned in producing it. Moreover, the mental association of iritis with a dyscrasia is not without its dangers. Notwithstanding great improvement in this respect in recent years, the diseases of the eye do not yet receive from medical students the full attention which is their due; and many young men go into practice with sufficiently clear notions about syphilis or rheumatism, but with very imperfect knowledge of the pathology and peculiar dangers of iritis. The rheumatism or the syphilis must not, indeed, be either overlooked or neglected; but, in the presence of iritis, the primary concern of the surgeon is with the eye. No amount or kind of constitutional treatment will prevent the formation of iritic adhesions; and an eye which recovers with adhesions, although it may for a time be restored to normal vision, and may seem quite well to the perceptions of the patient, is too often on the high-road to destruction. The adhesions are a frequent source of recurrence of the iritis, and each recurrence may give rise to fresh adhesions, until at last the whole of the pupillary margin is tied down to the capsule of the lens, and an impermeable barrier is interposed between the filtration area, already described in the papers on glaucoma, and the fluid constantly secreted by the ciliary processes. We then have what is, in effect, a secondary glaucoma, an accumulation behind the iris by which destructive pressure is exerted upon the retina, and by which, sooner or later, sight will be entirely destroyed. The treatment of iritis must be primarily directed to the prevention or to the detachment of adhesions, and only secondarily to the actual or supposed causes of the inflammation. We have first to think of the dangers which menace the eye, and afterwards of the general condition of the patient.

